# Body Caudal Undulation Measured by Soft Sensors and Emulated by Soft Artificial Muscles

**DOI:** 10.1093/icb/icab182

**Published:** 2021-08-20

**Authors:** Fabian Schwab, Elias T Lunsford, Taehwa Hong, Fabian Wiesemüller, Mirko Kovac, Yong-Lae Park, Otar Akanyeti, James C Liao, Ardian Jusufi

**Affiliations:** Locomotion in Biorobotic and Somatic Systems Group, Max Planck Institute for Intelligent Systems, Heisenbergstraße 3, 70569 Stuttgart, Germany; Department of Biology, Whitney Laboratory for Marine Bioscience, University of Florida, Saint Augustine, FL 32080, USA; Department of Mechanical Engineering, Seoul National University, Seoul 08826, Korea; Materials and Technology Center of Robotics, EMPA, Überlandstrasse 129, Zürich 8600, Switzerland; Aerial Robotics Lab (ARL), Department of Aeronautics, Imperial College London, South Kensington Campus, London SW7 2AZ, UK; Materials and Technology Center of Robotics, EMPA, Überlandstrasse 129, Zürich 8600, Switzerland; Aerial Robotics Lab (ARL), Department of Aeronautics, Imperial College London, South Kensington Campus, London SW7 2AZ, UK; Department of Mechanical Engineering, Seoul National University, Seoul 08826, Korea; Department of Biology, Whitney Laboratory for Marine Bioscience, University of Florida, Saint Augustine, FL 32080, USA; Department of Computer Science, Aberystwyth University, Aberystwyth, Ceredigion SY23 3FL, UK; Department of Biology, Whitney Laboratory for Marine Bioscience, University of Florida, Saint Augustine, FL 32080, USA; Locomotion in Biorobotic and Somatic Systems Group, Max Planck Institute for Intelligent Systems, Heisenbergstraße 3, 70569 Stuttgart, Germany

## Abstract

We propose the use of bio-inspired robotics equipped with soft sensor technologies to gain a better understanding of the mechanics and control of animal movement. Soft robotic systems can be used to generate new hypotheses and uncover fundamental principles underlying animal locomotion and sensory capabilities, which could subsequently be validated using living organisms. Physical models increasingly include lateral body movements, notably back and tail bending, which are necessary for horizontal plane undulation in model systems ranging from fish to amphibians and reptiles. We present a comparative study of the use of physical modeling in conjunction with soft robotics and integrated soft and hyperelastic sensors to monitor local pressures, enabling local feedback control, and discuss issues related to understanding the mechanics and control of undulatory locomotion. A parallel approach combining live animal data with biorobotic physical modeling promises to be beneficial for gaining a better understanding of systems in motion.

## Introduction

**Fig. 1:**
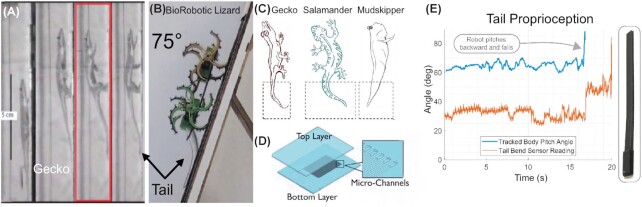
(**A**) Side view of a climbing gecko (*Hemidactylus platyurus*), showing the use of the tail to recover from a forefoot slip (Siddall et al. 2021a). (**B**) Climbing robot prototype with soft, sensor-integrated stabilizing tail able to climb up a slope of 75° (Siddall et al. 2021a). (**C**) Soft and stretchable strain sensors for comparative physiology and biomechanics study measure active tail responses (Souri et al. 2020). (**D**) Schematic soft stretch sensor, consisting of two layers fitted with micro-channels in between filled with eGaIn (Park et al. 2012). (**E**) Soft tail sensor is able to sense pitch-back in the robot (Siddall et al. 2021a)

Animals can modulate their movements in response to dynamic external disturbances and exhibit locomotion robustness via sensory feedback in conjunction with preflexes in large part due to the morphology and passive mechanics of their compliant structures as well as integrated sensing capabilities. These aspects enable them to adjust to unanticipated changes and enhance stability (e.g., Ghazi-Zahedi 2019, Siddall et al. 2021), thus providing potential for the design of more resilient robots traversing uneven terrain (e.g., Woodward and Sitti 2018). Tails have been shown to provide locomotion robustness in aerial, terrestrial and aquatic locomotion (Shield et al. 2021), such as body caudal fin swimming in fishes (Colgate and Lynch 2004; Crossin et al. 2004), twist feeding in crocodiles (Fish et al. 2007), and slip recovery (Fig. 1A) or water traversal by geckos (Nirody et al. 2018).

The research direction in biomimetic robotics has tended to be one-directional: nature’s features have been attempted to be replicated in detail in order to improve robot capabilities, with the goal of building robots with expanded locomotion efficacy across a range of environments. This research has provided a direct boost to the engineering capability, but only limited insight to the understanding of biological model system dynamics and control.

However, in recent years, roboticists and biologists are increasingly collaborating, gaining insights into biological behavior and mechanisms using physical robotic models approximating animal-like capabilities (Kovač 2013; Ijspeert 2014; Long et al. 2014). Physical models can offer powerful tools in the testing and refining of hypotheses on the evolution of locomotion patterns and appendages (McInroe et al. 2016; Nyakatura et al. 2019; Schultz et al. 2021). Exploration of biological features through the building of robophysical models can equip biologists with platforms that may help to gain insight of animal locomotion dynamics and control under conditions relevant to understanding of the model system, by providing a physical simulation tool, and without the need of observing the animal in a complex environment using complicated field techniques such as high-speed video capture (Bostwick and Prum 2003; Nirody et al. 2018; Yeaton et al. 2020) or on body sensors on animals which do not capture the body interaction with the environment (Aguilar et al. 2016; Gravish and Lauder 2018; Aydin et al. 2019). Such robots can be used to provide a systems-level approach to understand locomotion where the hardware (material and morphology) and software components (sensory and control) must work in unison to replicate movements that are seen in the wild. Furthermore, using robots offers the liberty to modify morphologies in a systematic way (larger parameter space), and perform movements that animals typically do not, or that can be dangerous for the animal.

The robotic, bio-inspired platforms have the advantage of selectively isolating and alternating interesting traits, and therefore providing a much larger parameter space for experimentation (Webb 2000). By simplifying the complex interactions found in living organisms, abstract physical robotic models may shed new light on the interdependence of various body parts to enable locomotion (Siddall et al. 2019), such as how climbing arboreal geckos employ an active tail reflex to dynamically respond to the loss of contact with a climbing surface (Fig. 1A, Siddall et al. 2021a), mechanisms used for mid-air reorientation (Fukushima et al. 2021; Siddall et al. 2021b), or the study of undulatory movements produced by a single actuation point located posterior to the head of a physical robotic model (Akanyeti et al. 2016). Additionally, physical models capture the relationship between body mechanics and coupled environmental effects, for example, the fluid dynamics of a swimming fish. Investigating the fluid-body interaction in live specimens is often is often time-consuming, as the animal’s behavior is frequently unpredictable.

Another advantage of physical models over their living equivalents is that they allow us to investigate the motion patterns that are used for effective and optimized locomotion and which forms of motion are required to support a living organism. For example, fish must maintain a water flow through their gills in order to provide oxygen to their tissue (Akanyeti et al. 2016). Physical models are inherently devoid of such living needs, and it is possible to investigate the characteristics that are actually and exclusively advantageous for the locomotion. Similarly to how experimental observation in biology enables advancements in robotics, bio-inspired robotics expands the possibilities for testing biological hypotheses.

The main motivation for this paper is to encourage the increased utilization of multidisciplinary approaches lending from soft robotic physical modelling and multi-scale manufacturing techniques between biologists, materials scientists, and engineers as well as hypothesis formulation from the outset, for example with respect to how undulatory locomotion might be affected by tail sensory feedback. Biorobotic models increasingly include lateral body motions, particularly back and tail bending, which is crucial for permitting undulation in fishes, amphibians, and reptiles (e.g., Thandiackal et al. 2021). We survey the use of physical modeling in conjunction with soft robotics and sensors to monitor local pressures, enable local feedback control, and address themes pertaining to insight the mechanics and control of undulatory locomotion (e.g., Kim et al. 2020; Tytell and Long 2021; Thandiackal et al. 2021). Biorobotics could also be used in a broader context of the comparative method, complementing the understanding of locomotion performance of extinct and extant species (e.g., Crofts et al. 2019) or even as machines that eventually can be deployed for future space missions (e.g Ng and Lum 2021). The ability to observe physical systems in action would constitute a substantial benefit in complex circumstances where it may be difficult to examine them through live animal experimentation. A complementary approach investigating live animal data in tandem with biorobotic physical modeling would be desirable for greater understanding of systems in motion. To achieve this it would be advisable to develop interdisciplinary projects jointly from the outset, with the goal of refining hypothesis testing and possible material or device development (e.g., Miriyev and Kovač 2020). While computational advances have steadily enhanced artificial intelligence, the physical capabilities of robots have not been improved in the same measure. Biomimetics and robotics with more life-like capabilities (e.g., Fish 2020; Triantafyllou et al. 2020) could help accelerate locomotion capabilities of robots by offering a different perspective.

In this paper, we examine soft sensors and biorobotics to better understand various forms of body caudal undulations. We aim to show the capabilities and possibilities of these novel soft sensors, and their potential together with soft robots for verification and exploration of locomotion traits used by living organisms. Extending on the practice of using flexible but passive structures (as seen in Lauder et al. 2012), this paper puts the focus on active soft robotic testing platforms with incorporated soft sensing abilities. We propose that soft sensors and actuators are the next major push to enable robots to become more life-like in their capability, so that they may better emulate how animals move. Seeing that tails have many more degrees of freedom than other limbs, soft actuators are much better suited than conventional ones.

## Biorobotic physical models made of soft active materials

**Fig. 2:**
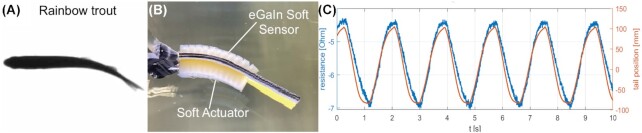
Flow tank experiments with a soft robotic fish. (**A**) Ventral view of the Bluegill Sunfish (Liao et al. 2003). (**B**) Top view of soft robotic fish with soft actuator and sensor during undulation locomotion (Lin et al. 2021) (**C**) Comparison of strain sensor readings and undulation tail position read from video analysis in a recirculating flow tank

To gain insight in the underlying processes of tail reflexes and passive dynamics, one can integrate stretchable strain sensors into active soft robotic platforms for sensory feedback (Fig. 2B). Stretchable strain sensors have the potential to provide reliable sensory feedback that can yield robust capabilities. When intertwined with a soft actuator, the sensor can provide crucial information about traits that make efficient undulatory locomotion possible (Wright et al. 2019).

Animal strategies such as tail usage during climbing (Fig. 1A) or body stiffening during swimming (Fig. 2A) can be replicated and studied using abstract active robotic platforms.

The active component of the robotic platform is important for understanding how a biological organism interacts with its surroundings. Only then can one confidently assert that the platform mimics the biological species, not just in terms of size and mass, but also in terms of material structure, mechanical composition, and compliance.

The gecko (*Hemidactylus platyurus*) uses its tail to recover from forefoot slip (Fig. 1A). Inspired by that, a BioRobotic Lizard with a fully flexible, soft-sensor integrated tail was built (Fig. 1B) to explore the effect of the tail for incline climbing and obstacle traversal performance in rapid locomotion (Siddall et al. 2021a).

First, climbing trials were carried out without the robotic tail. The robot was unable to ascent at an angle greater than 45° without the tail to maintain forefoot touch. With the tail attached, in contact with the surface, and with a slight preload added, this angle increased to 80°, and changes from the horizontal to inclines of up to 75° became possible (Fig. 1B, Siddall et al. 2021a). The BioRobotic Lizard platform gives us the possibility to do a full climbing angle parameter sweep, and measure exactly at what point the tail becomes essential for the ability to ascend.

Figure 1D shows the measurements of the soft sensors integrated in the BioRobotic Lizard’s tail during situation where the robot slips during an ascend of 70°. By incorporating a controller, we envision to actively respond to the sensor measurements of the slippage and catch the fall, resembling the active tail reflex to dynamically respond to the loss of contact with the climbing surface of geckos (Fig. 1A).

A robotic platform consisting of a soft robotic fish with a backbone stiffness comparable to Bluegill Sunfish (developed in Jusufi et al. 2017; Wright et al. 2019; Wolf et al. 2020) was expanded to investigate closed loop responses via soft sensors in both air and water with various undulation frequencies to demonstrate the proprioceptive sensing skills required to emulate more life-like swimming with undulatory shape changes on the body (Lin et al. 2021; Fig. 2A and B). Fish modulate an inherent cycle to preserve the necessary speed by varying the ratio of bursting to coasting while keeping the cycle length approximately constant (Li et al. 2021). Beyond closing the loop with feedback in non-moving fluids, the next step consists of experimental validation of the closed loop controller at different water flow speeds in a recirculating flow tank (Fig. 2C).

**Fig. 3:**
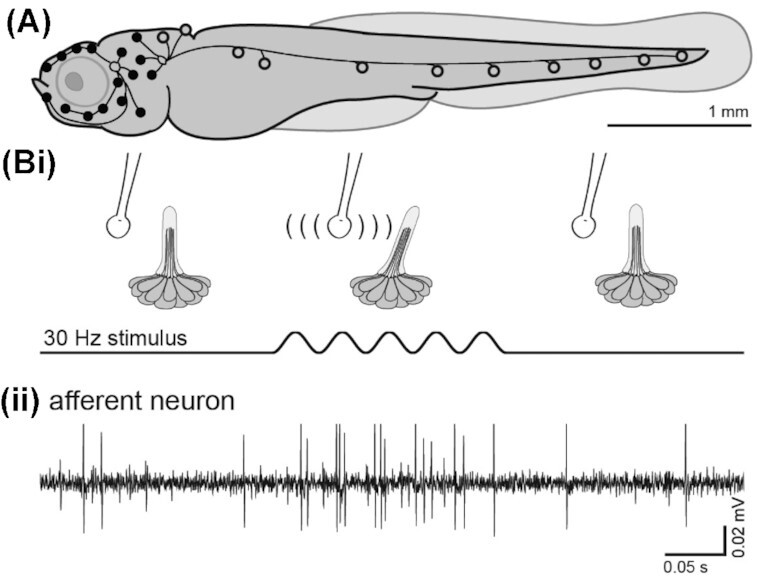
(**A**) Schematic of anterior (solid) and posterior (hollow) lateral line sensor position on a larval zebrafish (5 days post fertilization). (**B**) (i) Deflection of the neuromast cupula stimulates the hair cells which elicits an (ii) evoked response observed via electrophysiological recording of a posterior lateral line afferent neuron

In fishes, the flow-sensitive lateral line organ is comprised of a functional unit, the neuromast, which covers the head and body and lies on or near the skin surface. A neuromast is composed of a hair-like bundle that is embedded into a gelatinous cupula, which deflects when fluid moves relative to the body (Fig. 3B). This triggers a mechanotransduction event in which signals from the fluid environment are translated into electric nerve signals. These signals are then transmitted to the brain via afferent neurons. Using transparent, larval zebrafish (Fig. 3A), we can target afferent neurons and record their electrical signals in response to controlled mechanical deflections of a single neuromast lying on the body surface. These superficial neuromasts are sensitive to flow velocity. Neuromast deflections are generated using a fine glass pipette controlled by piezoelectric transducer, which can impose sine wave frequencies that match the tailbeat frequencies of freely swimming zebrafish. In adult fish, neuromasts are recessed into canals in the scales, and this mechanical filter makes them sensitive to pressure (McHenry and Liao 2014).

It has been suggested that lateral-line activity during undulatory body motions is an important feedback link in closed-loop control of fish swimming (Roberts 1969; Roberts and Russell 1972; Ayali et al. 2009). Similar feedback signals have been found in the spinal cord as well (Böhm et al. 2016; Picton et al. 2021).

The soft sensory output of the robotic fish with a feedback controller is evaluated, and we show, that the sensors are fully capable of capturing the deflection of the fish in real-time (Fig. 2C). This fish-inspired robotic platform allows for the discovery of the effect of tail stiffness and undulation frequency on thrust generation and with novel soft sensors can sense its environment as well as respond to perturbations. Compared to a natural fish (Fig. 2A), the platform has the ability to test different frequencies and co-contractions at will and test parameters that would not be possible to find in nature.

Fish are able to modulate body and fin stiffness (Long Jr 1998; Lauder et al. 2012), and exploit vortices found in turbulent flows (Liao et al. 2003) to increase swimming efficiency. Experiments with physical, fish-inspired platform suggest that the timing (measured as phase difference) between yaw and side-to-side movements of the head may be key in achieving better swimming performance during steady (Akanyeti et al. 2016) and unsteady swimming (Long Jr et al. 2002; Akanyeti et al. 2017). The internal dynamics and the mechanisms through which soft structures are exploited for attaining this remarkable propulsive efficiency are under-explored.

**Fig. 4:**
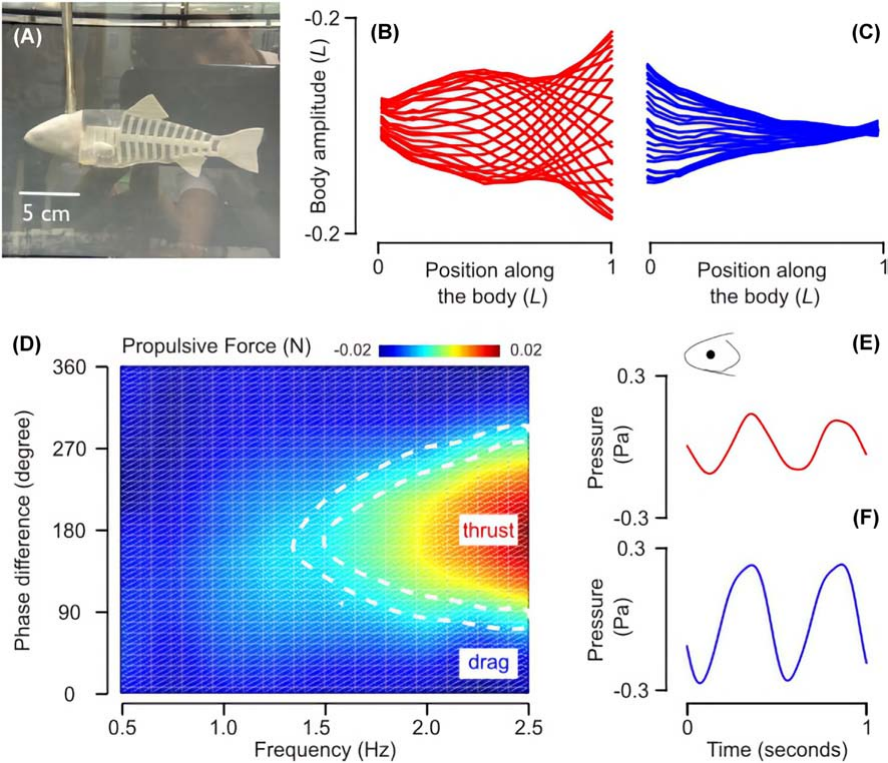
(**A**) A soft physical model inspired from a rainbow trout. (**B**) Midline reconstructions of the model for one tailbeat cycle show the body amplitude envelope for thrust-producing kinematics, which are similar to a live trout during steady swimming. (**C**) The amplitude envelope for drag-producing kinematics is very different from the kinematics of a real trout. (**D**) Heat map showing average propulsive force produced over one tailbeat cycle as a function of simultaneous heave and yaw movements; horizontal axis: oscillation frequency and vertical axis: timing between heave and yaw movements (0° and 180° indicates in-phase and out-of-phase movements, respectively). Regions with positive (red) and negative (dark blue) values show thrust and drag producing parameters, respectively. Note the region in between the two dashed lines (light blue) show the self-propelled speed where thrust is equal to drag. (**E and F**) A single pressure sensor placed on the side of the head near the eye (inset diagram) is enough to reveal that unique pressure profiles exist during thrust and drag-producing swimming motions shown in (B) and (C)

To address these unknown parameters, we performed experiments with an additional submersible robotic platform, inspired by the rainbow trout, and integrated with pressure sensors. This time, a three-dimensional (3D) CAD model from scanned images of a specimen was designed and a bio-inspired physical model, consisting of a rigid head, flexible backbone, and a soft body was constructed (Fig. 4A) and tested. From a single actuation point located posterior to the head, undulatory movements were produced. This allowed us to assess performance in terms of thrust generation and swimming kinematics.

At first sight, the comparison between thrust (Fig. 4B) and drag-producing kinematics (Fig. 4C) suggest that there are favorable head movements that allow the physical model to adopt midline kinematics similar to fish and increase swimming efficiency. However, further comparison between the model and fish kinematics is needed to quantify similarity more objectively (Fetherstonhaugh et al. 2021). Our results are just a proof-of-concept demonstrating that pressure profiles around the head can be linked to propulsive performance. We recognize that propulsive performance of a model may vary depending on the swimming speed and geometry and size of the physical model (for instance, see Piñeirua et al. 2017), which warrants further investigation.

Systematic analysis on how thrust production varies as a function of oscillation frequency and phase difference (Fig. 4D) suggest that by controlling the timing of head movements, the physical model can choose between slowing down (by generating drag), speeding up (by generating thrust), or swim steadily for the same flow conditions. In addition, we managed to reveal unique pressure profiles for these thrust and drag-producing swimming motions (Fig. 4E and F), suggesting that flow-relative sensory feedback can be used to enhance swimming performance.

With recent research indicating that internal mechanical sensors may be used to determine tail beat amplitude and frequency (Sánchez-Rodríguez et al. 2021), our work on pressure sensors that detect changes in the external environment may be considered important to assure function reliability and robustness. Thus, both internal and exterior sensors are likely critical for optimizing tailbeat amplitude and frequency selection.

## Soft sensors

**Fig. 5:**
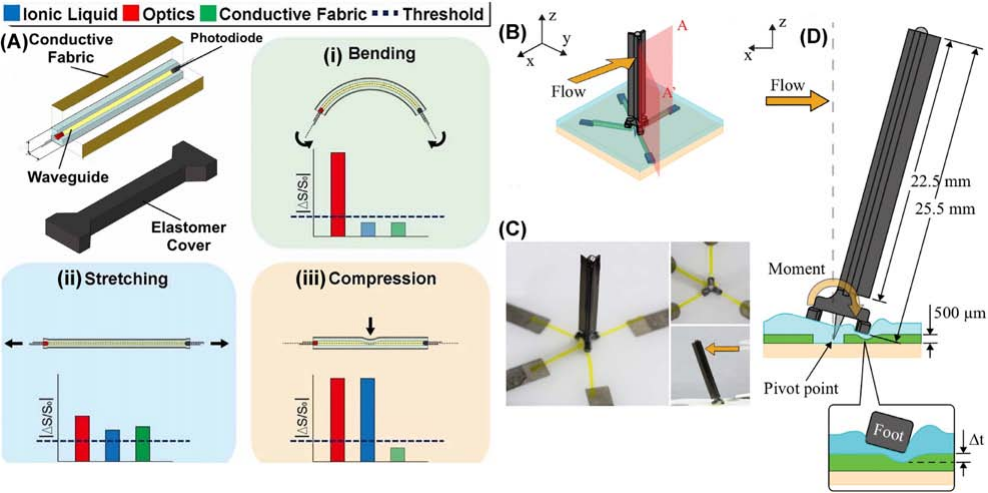
Examples of liquid-embedded soft sensors. A multifunctional soft sensor with a combination of microfluidic, optical, and piezoresistive sensing mechanisms (Kim et al. 2020; left) and a biomimetic flow sensor composed of ionogel microchannels and a hair-like structure (Shin et al. 2019; right)

Wearable and skin-mounted sensors may help in the creation of robophysical models by offering insight into the mechanics and control (e.g., tailed model systems in Fig. 1C, reproduced from Souri et al. 2020, modified) of locomotion, including and passive mechanics, as well as potentially explaining how an organism’s muscles, sensors, motor pattern generators, and brain interact to produce coordinated movement in response to unexpected perturbations (e.g., Nishikawa et al. 2007). We envision a robotic fish platform equipped with arrays of multi-functional soft sensors (Fig. 5-left) for proprioception and flow sensors (Fig. 5-right) for mechanoreception. Elastic and viscoelastic properties are critical factors for the material selection of soft robotic components in order to adapt the mechanical flexibility and multifunctionality intrinsic to natural organisms (Banerjee et al. 2021).

Capacitive soft sensors are made out of conductive materials, and change their capacitance between the two deformable layers when deformed (Ponce Wong et al. 2012; Atalay et al. 2018; Li et al. 2016; Kim et al. 2019). Other kinds of soft sensors are based on optical properties, such as light intensity (Larson et al. 2016; To et al. 2018; Xu et al. 2019a; Jung et al. 2020) and different wavelengths (Xu et al. 2018; Zhuang et al. 2018).

Resistive soft sensors transduce external mechanical stimuli into electrical signals. Although various sensors of this type based on different mechanisms have been proposed, such as conductive polymers (Wang et al. 2017), conductive fabrics (Atalay et al. 2018), and thin-metal-film coated polymers (Firouzeh and Paik 2015), we focus on a particular class of resistive soft sensors made of highly deformable elastomer embedded with conductive liquids, where the electric resistance of liquid-filled microchannels in an elastomer matrix changes when the matrix structure is stretched, compressed, or bent (Park et al. 2010; Majidi et al. 2011; Park et al. 2012; Vogt et al. 2013; Shin et al. 2020; Fig. 5A–C).

These sensors are extremely useful in soft robotics due to their high sensitivity, stretchability, and ability to deform dynamically. Since the main sensing medium is a liquid that can be easily formed in any shape without limiting or degrading the existing range of motion and degrees of freedom of the host structures, these sensors can be easily attached to or embedded in any body parts of a soft robot. This makes them especially suitable for measuring movements that are inspired by animal locomotion and performed by soft robotic platforms and to detect contacts made by external environments, as demonstrated by artificial proprioceptors (Wirekoh et al. 2019; Park et al. 2020) and mechanoreceptors (Kim et al. 2018; Shin et al. 2019), respectively.

One of the most commonly used conductive liquids in the sensors is eutectic gallium-indium (eGaIn; Chiechi et al. 2007; Dickey et al. 2008), a metal alloy that maintains a liquid state at room temperature. The high electrical conductivity makes it ideal for stretchable sensors and circuits when embedded in elastomer. Moreover, the high surface tension and viscosity makes the material not only easy to be injected but also stable in microchannels even with large deformations.

Although eGaIn is known to be nontoxic unlike mercury, its safety has not been fully investigated on the absorption by humans. If biocompatibility or optical clarity are needed in the system, an alternative to liquid metals is ionic liquid. Ionic liquids, for example saline solutions, are biocompatible but still electrically conductive, and can be used as a replacement of eGaIn for soft sensors (Chossat et al. 2013, 2015). Moreover, the optical transparency of ionic liquid makes it possible to use the liquid-filled microchannel as an optical waveguide for additional functionalities (Kim et al. 2020).

## Bio-inspiration for multi-modal sensing

If multiple sensing mechanisms can be integrated in a single sensor structure, multi-modality can be accomplished easily while retaining design and fabrication simplicity. Examples of it can again be found in biology, e.g., on a larval zebrafish (Fig. 3). Sensory neurons called low-threshold mechanoreceptors (LTMRs) are one type of the mechanoreceptors connected to hair follicles, which detect physical stimuli applied to the skin. It is known that the LTMRs are functionally distinct and triggered by particular types of physical stimuli (Abraira and Ginty 2013). In detail, the LTMRs of the hairy skin are categorized into three subtypes Aβ-, Aδ-, and C-LTMRs, which are distributed around follicles of three different types of hairs, such as guard, zigzag, and awl/auchene hairs, as a form of a bundle. The bundles at the follicles are composed of different combinations of the subtypes of the LTMRs. When a physical stimulus is applied to the skin, it excites the bundles by moving hairs on the skin, and the bundles generate electrical signals.

**Fig. 6:**
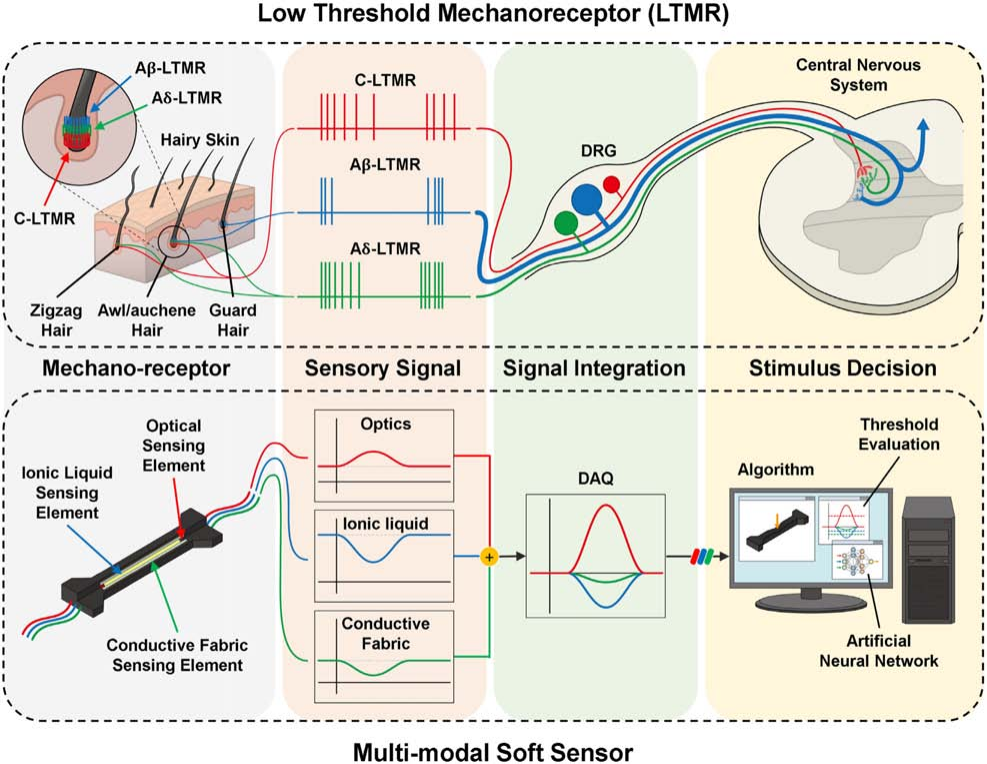
Comparison between innocuous touch sensing of hairy skin using low-threshold mechanoreceptors and proposed soft multi-modal sensing

By interpreting the distinct electrical signals activated by the LTMR combinations, the central nervous system can distinguish the difference of the stimuli near the skin, such as pressure, friction, or even both. As depicted in Fig. 6, the multi-functional soft sensor is available to detect three different deformation modes, bending, compressing, and stretching, which can be interpreted based on the signals from the three sensing mechanisms. By integrating the information from the three signals, the unique pattern can be determined. The presented multi-functional sensor is capable of not only detecting three individual deformations but also decoupling combined ones.

Since robotic platforms for robotic-inspired biology normally operate in unstructured environments, e.g., water or rough terrain, there will be various information from both the inside and the outside of the body, such as bending angle, thrust, pressure, and drag force, useful for controlling the robot with stability and safety. The multi-functioning sensor mimics the function of animal LTMRs, which can decompose multiple mixed signals while sharing a single structure skin. Therefore, acquiring various sensor signals can be important in this study, which can be achieved through different types of soft sensors distributed in the body.

## Discussion and outlook

In this article, we present soft sensors for soft robotic platforms to investigate body caudal undulation locomotion and tail usage.

By combining bio-inspired soft robotic platforms with integrated sensing capabilities, we are able to perform systematic experimental validation of tail and undulation locomotion concepts that would be extremely difficult to execute in living organisms. Environmental variables can be controlled to obtain repeatable outcomes and by abstracting complex actions to single traits, the influence of the tail can be measured separately and its importance evaluated.

The comparison in swimming performance of our soft fish model compared to fishes shows that our fish has a greater ratio of lateral to thrust force generation (nearly 5:1), whereas fishes swimming with an undulatory body wave typically show a 2:1 ratio (see Lauder et al. 2012). This indicates that our model is most likely a substantially lesser efficient swimmer than swimming fish like mackerel or bluegill. We would like to investigate this in the future and propose using particle image velocimetry to compare the fish’s swimming performance with a soft robot of similar size.

Beyond comparative biomechanics research, applications of stretchable sensors could potentially extend to include diagnostics. More detailed artificial lateral line analysis based on variable stiffness, soft sensing feedback (e.g., soft strain or pressure sensor) can be done in the future. We envision to adapt the sensors and place them on real animals, to revolutionize the way we can measure locomotion, movements and muscle activity.

In addition, shape sensors could be relevant for identifying morphological changes for impulsive and dynamic aquatic locomotion, such as in squid where it has been shown that changes in body cross-section can lead to thrust peaks of up to a factor of 2.6 (Steele et al. 2017). Similarly, flying fish exhibit very fast undulation of their tail for aquatic escape (with speeds of up to 10–20 m/s (Park and Choi 2010)). This shape morphing happens relatively quickly (in less than 1 s) and will require appropriately matched sensor dynamics to measure the time-independent changes in body shape. It has been shown that microfluidic soft sensors can detect a sinusoidal input of 20% strain with a response time of less than 0.1 s and up to 5 Hz. Therefore, they could potentially be used for such fast motions although dynamic effects on changing fluid forces during the motion could potentially influence the measurements (Xu et al. 2019b). Future work could target tailored methods for high-speed proprioception and closed-loop control for fast-moving aquatic systems where the environmental interaction and forces are unsteady and dynamic.

We intend to do more study on the effect of active body stiffness adjustment on fish swimming speed and performance. It is assumed that, regardless of the hydrodynamic load, the body’s normal driving frequency decreases the force needed to induce a given motion.

The robotic platforms can be developed further; a scaled-down version of the soft fish, disturbance rejection for situational awareness and obstacle clearance with sensory feedback (e.g., soft strain or pressure sensor), or soft sensor integration for flow sensing with velocity and angular sensitivity are only a few of the many possibilities for using physical robotic platforms to gain insight into biological processes.

Ultimately, new insights into behavior, neuromuscular regulation, and mechanosensory receptivity can be obtained by recording the biological activity of animals’ caudal appendages using soft robotic systems equipped with advanced soft sensing capabilities. Our approach demonstrates that bio-inspired robots are effective tools for investigating natural performance.

## Supplementary Material

icab182_Supplemental_Files

## Data Availability

No new data were generated or analyzed in support of this research.

## Notes

From the symposium “An Evolutionary Tail: Evo-Devo, Structure, and Function of Post-Anal Appendages” presented at the annual meeting of the Society for Integrative and Comparative Biology virtual annual meeting, January 3–February 28, 2021.
