# Supplementary figures and images for: Body Caudal Undulation Measured by Soft Sensors and Emulated by Soft Artificial Muscles
Source: Integr Comp Biol. 2021 Aug 20;61(5):1955–65. doi: 10.1093/icb/icab182 (PMC8699111; doi:10.1093/icb/icab182)

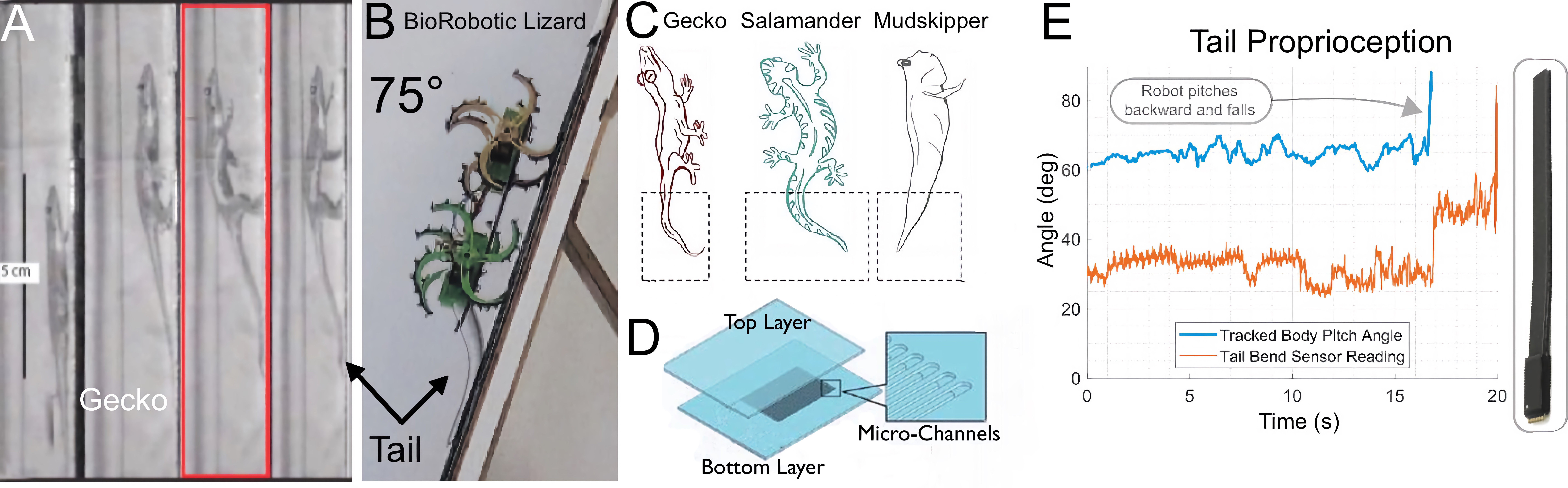

Supplement: icab182_Supplemental_Files [file icab182_supplemental_files.zip › icb-2021-0185-File003.jpg]

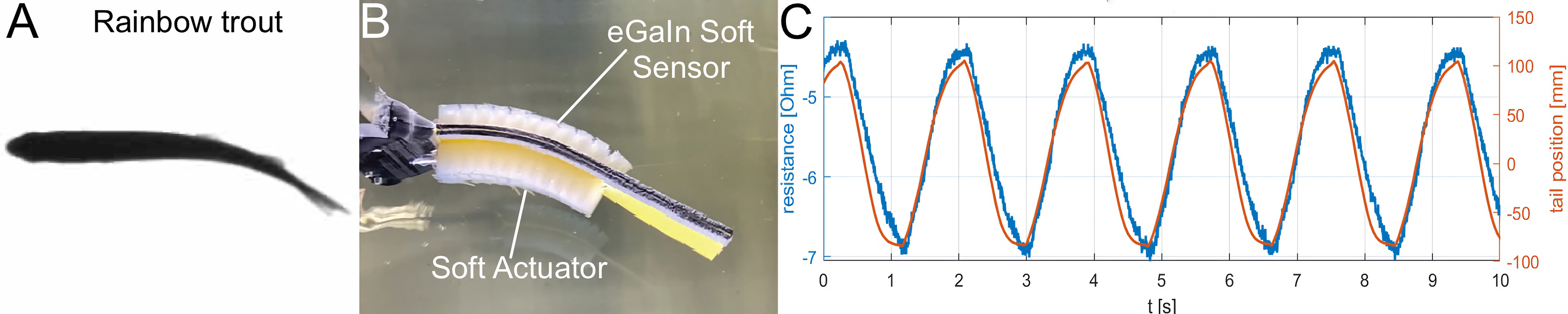

Supplement: icab182_Supplemental_Files [file icab182_supplemental_files.zip › icb-2021-0185-File004.jpg]

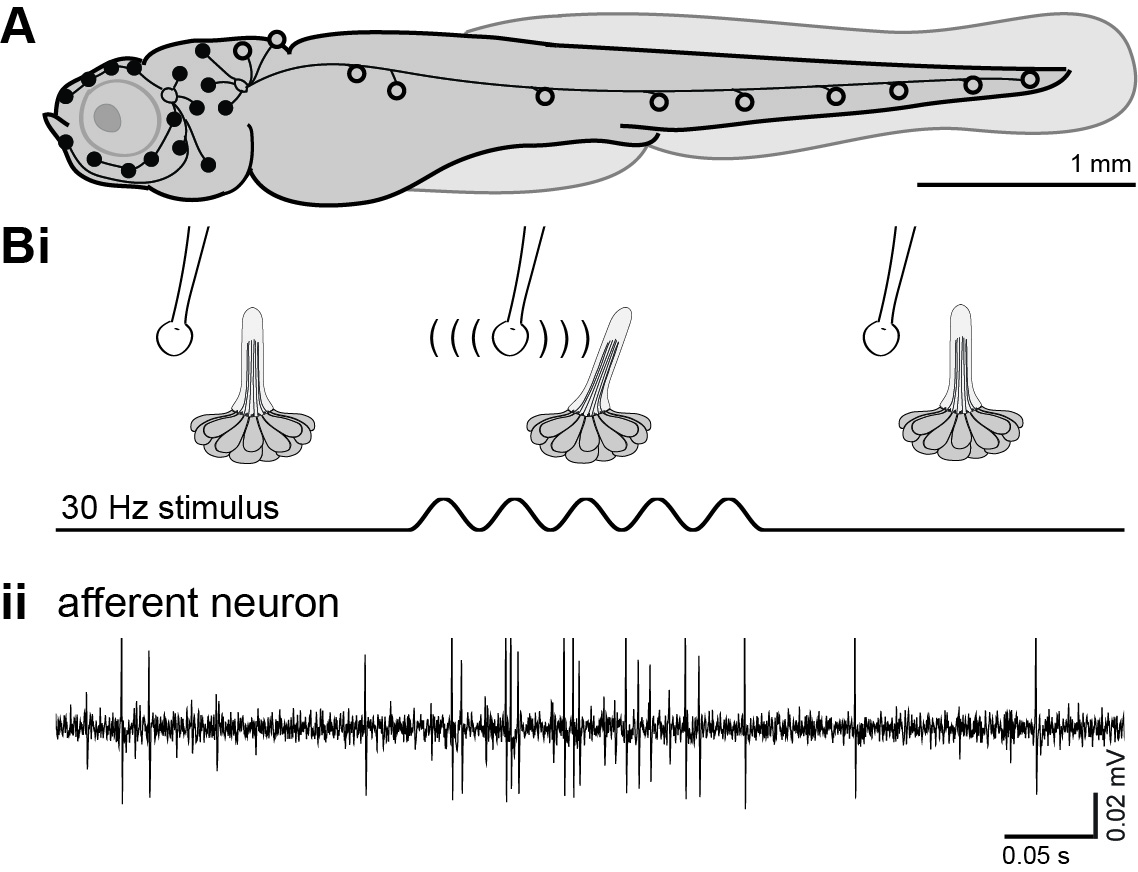

Supplement: icab182_Supplemental_Files [file icab182_supplemental_files.zip › icb-2021-0185-File005.jpg]

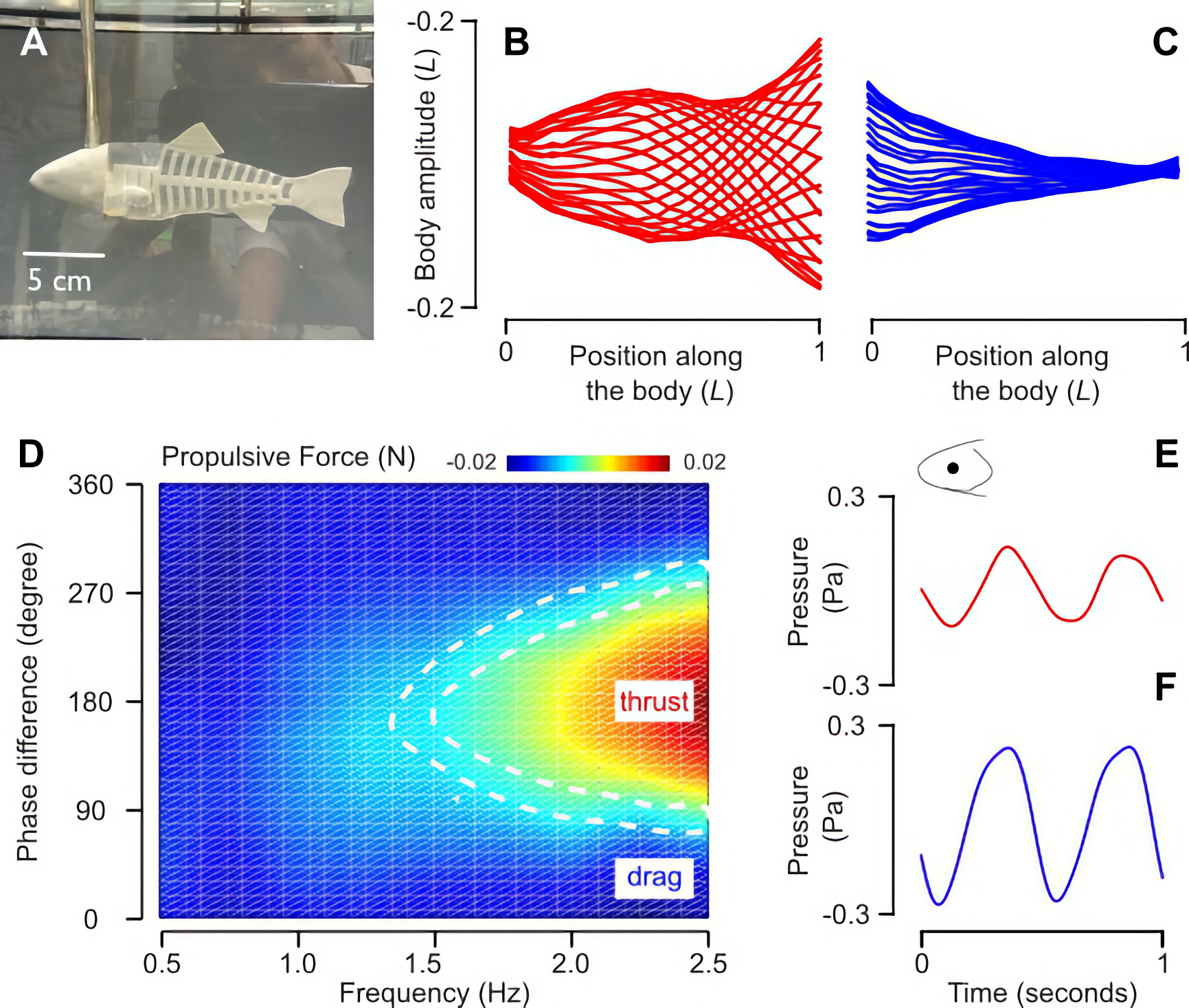

Supplement: icab182_Supplemental_Files [file icab182_supplemental_files.zip › icb-2021-0185-File006.jpg]

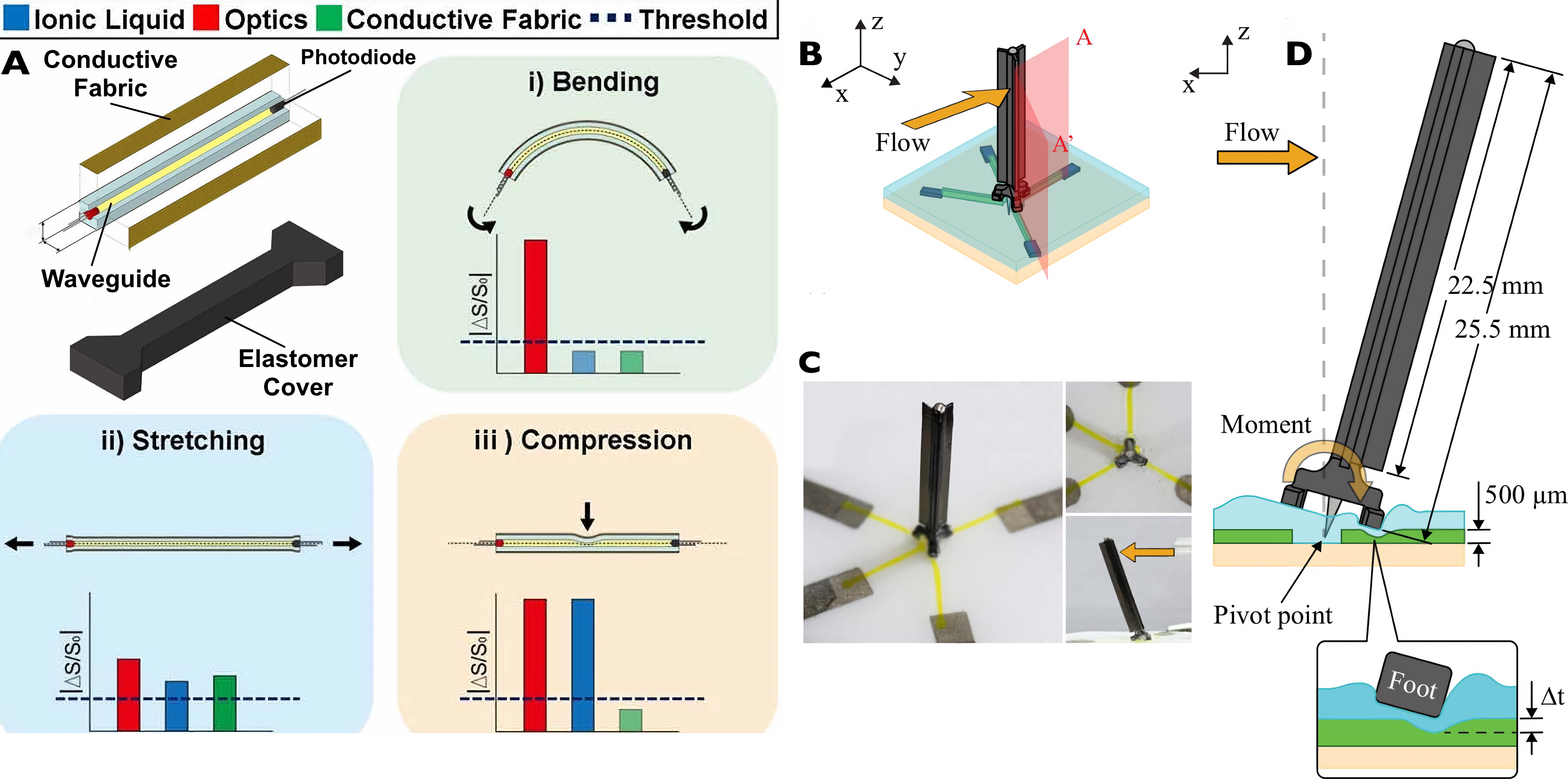

Supplement: icab182_Supplemental_Files [file icab182_supplemental_files.zip › icb-2021-0185-File007.jpg]

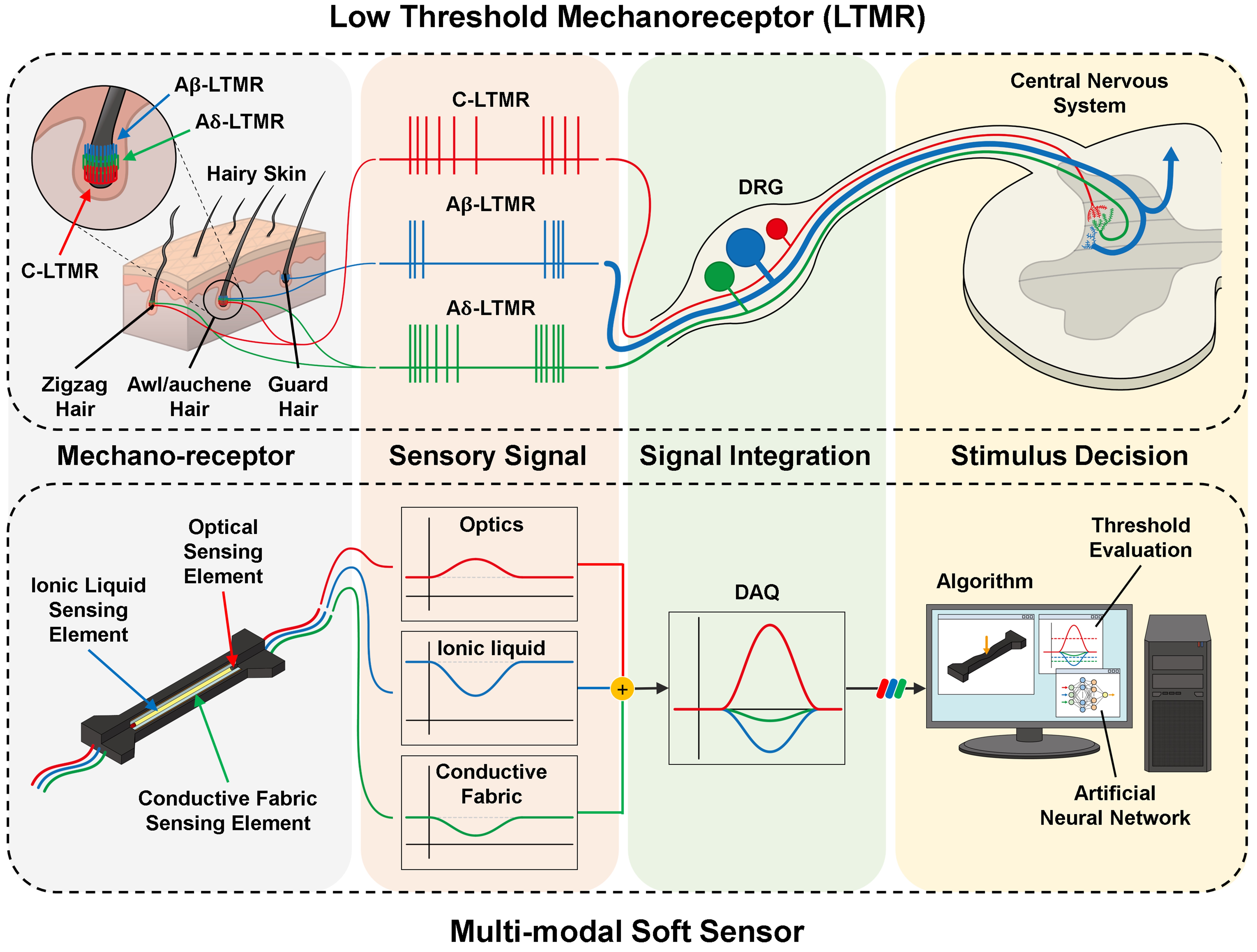

Supplement: icab182_Supplemental_Files [file icab182_supplemental_files.zip › icb-2021-0185-File008.jpg]
